# High prevalence of pulmonary tuberculosis among female sex workers, men who have sex with men, and transgender women in Papua New Guinea

**DOI:** 10.1186/s41182-020-00293-w

**Published:** 2021-01-13

**Authors:** Barne Willie, Avi J. Hakim, Steven G. Badman, Damian Weikum, Rebecca Narokobi, Kelsey Coy, Josephine Gabuzzi, Simon Pekon, Samson Gene, Angelyn Amos, Martha Kupul, Parker Hou, Nick M. Dala, David M. Whiley, Johanna Wapling, John M. Kaldor, Andrew J. Vallely, Angela Kelly-Hanku

**Affiliations:** 1 Papua New Guinea Institute of Medical Research, Goroka, 441 EHP Papua New Guinea; 2 US Centers for Disease Control and Prevention, Atlanta, USA; 3 Kirby Institute for Infection and Immunity, UNSW Sydney, Sydney, Australia; 4 Papua New Guinea National Department of Health, Port Moresby, Papua New Guinea; 5 Centre for Clinical Research, University of Queensland, Brisbane, Australia

**Keywords:** Tuberculosis, Female sex workers, Men who have sex with men, Transgender women, HIV

## Abstract

**Background:**

Papua New Guinea (PNG) has a tuberculosis (TB) case notification rate of 333 cases per 100,000 population in 2016 and is one of the 14 countries classified by the World Health Organization (WHO) as “high-burden” for TB, multi-drug-resistant TB (MDR-TB), and TB/HIV. HIV epidemic is mixed with a higher prevalence among key populations, female sex workers (FSW), men who have sex with men (MSM), and transgender women (TGW).

**Methods:**

We conducted a cross-sectional HIV biobehavioral survey (BBS) using respondent-driven sampling method among FSW, MSM, and TGW in Port Moresby, Lae, and Mt. Hagen (2016–2017). As part of the study, participants were screened for the four symptoms suggestive of TB infection using the WHO TB screening algorithm. Sputum and venous whole blood samples were collected and tested for pulmonary TB and HIV infection, respectively. Pulmonary TB testing was performed using GeneXpert®MTB/RIF molecular point-of-care test, and HIV testing was done following the PNG national HIV testing algorithm. All data discussed are weighted unless otherwise mentioned.

**Results:**

Among FSW, 72.6%, 52.0%, and 52.9% in Port Moresby, Lae, and Mt. Hagen, respectively, experienced at least one symptom suggestive of TB infection. Among MSM and TGW, 69% and 52.6% in Port Moresby and Lae, respectively, experienced at least one symptom suggestive of TB infection. Based on GeneXpert®MTB/RIF results, the estimated TB prevalence rate among FSW was 1200, 700, and 200 per 100,000 in Port Moresby, Lae, and Mt. Hagen, respectively. Among MSM and TGW, the estimated TB prevalence rate was 1000 and 1200 per 100,000 in Port Moresby and Lae, respectively. Co-prevalence of TB/HIV among FSW was 0.1% in Port Moresby and 0.2% in Lae. There were no co-prevalent cases among FSW in Mt. Hagen or among MSM and TGW in Port Moresby and Lae.

**Conclusions:**

Key populations have a higher estimated rate of pulmonary TB than the national rate of pulmonary and extra-pulmonary TB combined. This showed that screening key populations for TB should be integrated into HIV programs regardless of HIV status in PNG’s national TB response.

**Supplementary Information:**

The online version contains supplementary material available at 10.1186/s41182-020-00293-w.

## Introduction

In 2018, an estimated 10 million people worldwide had tuberculosis (TB) with 1.5 million deaths attributable to the disease [1]. Infection with HIV is one of the main predisposing factors for TB infection (TB acquisition and development). People living with HIV (PLHIV) are most at risk of TB infection, and they are reported to have the highest TB-related deaths (17%, 251,000 deaths out of 900,000 PLHIV) in 2018 [1]. HIV key population such as prisoners, people who inject drugs, female sex workers (FSW), men who have sex with men (MSM), and transgender women (TGW) [2] are also identified to be at greater risk of TB infection because of their risk for HIV infection and/or the settings to which they are in (prison jails for example) that allow for easy acquisition of TB [2]. To respond more effectively to the dual burden of HIV and TB, the revised international guidelines for HIV biobehavioral surveillance (BBS) among key populations recommend in settings with a high TB prevalence biological testing be expanded to include screening and testing for TB [2, 3].

To date, the majority of the epidemiological TB research among HIV key populations has been focused almost exclusively on prisoners and people who inject drugs [4–11]. The limited TB data among FSW, MSM [12], and TGW showed that there is a concerning gap in TB epidemiological knowledge, particularly in settings similar to Papua New Guinea (PNG) with high TB prevalence and concentrated HIV epidemics.

PNG is the most populated (8.7 million people) Pacific Island nation and has a mixed HIV epidemic with prevalence disproportionately higher among key population. In 2010, the estimated HIV prevalence among FSW was 11 times higher (11.8%) [13] when compared to the general population prevalence of 0.9% [14]. The case notification rate for all forms of TB in PNG was reported at 333 cases per 100,000 population in 2016 [15].

PNG is one of only 14 countries classified by the World Health Organization (WHO) as having a high burden of TB, MDR-TB, and TB/HIV [1], and there is no published data on TB prevalence among HIV key population. As part of this BBS study, TB testing on sputum samples was done to determine pulmonary TB, rifampicin-resistant TB, and TB/HIV prevalence among FSW and MSM and TGW in the three largest cities of PNG.

## Study population and methods

We used respondent-driven sampling (RDS) to recruit FSW and MSM and TGW in Port Moresby (National Capital District) from June to October 2016, in Lae (Morobe Province) from January to June 2017, and in Mt. Hagen (Western Highlands Province) from August to December 2017. RDS is a variant of snowball sampling used to produce sampling weights and approximate a random sample [16, 17]. Detailed description of the methods used in this study can be found in these references [18–20].

### Inclusion criteria

To be eligible to participate in the BBS, participants had to be ≥ 12 years of age, could speak English or Pidgin, and have a valid study recruitment card known as a coupon. For FSW, they had to be born a female and have sold or exchanged sex for money, goods, or services in the past 6 months, while for MSM and TGW, they had to be born a male and have engaged in oral or anal sex with a person born male in the past 6 months.

The age of inclusion reflects PNG HIV-related law that states that a person 12 years or older can access health service for HIV testing without parental or guardian consent [14]. Since the study was designed to determine HIV prevalence and estimate population size, the time frame of 6 months was used to cater for the window period of HIV initial infection to the time of sero-conversion. Staff were trained to identify and refer all sexually exploited persons including those under the age of 18 years to partner organizations experienced in providing counseling, health, social, and other protective services to these populations.

### Data collection

At enrolment in the BBS, eligible participants provided verbal informed consent for STI (including HIV) and TB testing. Venous whole blood was collected for HIV testing and sputum for pulmonary TB testing. Since we were unaware of participants’ HIV status at the time of TB screening, we used the more sensitive WHO TB screening algorithm for PLHIV [21]. This algorithm asks for four symptoms that are suggestive of TB infection: unexplained weight loss, cough, fever, or having night sweats in the last 2 weeks [21].

Participants who reported experiencing one or more of the four symptoms were deemed eligible for TB testing and were asked to provide sputum sample for pulmonary TB testing using GeneXpert MTB/RIF® (Cepheid, Sunnyvale, CA, USA) molecular point-of-care test. Those unable to produce sputum were referred to a local TB health facility for chest X-ray. Participants who returned a positive TB test result were provided with an anonymized referral form detailing their results (including if the result indicated rifampicin resistance) with same day escorted referral to a TB clinic in the study city for further treatment and care.

HIV testing was done following the PNG national HIV testing algorithm by performing a primary screening test using Determine HIV-1/2 antibody-antigen test kit (Alere, Hannover, Germany) followed by a confirmatory test using Stat-Pak HIV-1/2 test kit (Chembio, New York, NY, USA).

In this study, prevalence rates for TB and HIV are defined as the weighted proportion of the number of people tested positive for pulmonary TB and/or HIV in the given key population while TB and HIV co-infection is defined as the weighted proportion of the number of people who tested positive for pulmonary TB in the population and who also tested positive for HIV.

### Data analysis

Data analysis was conducted using RDS-Analyst (Los Angeles, CA, USA) version 5.7 using Gile’s Successive Sampling estimator to develop weighted population estimates and 95% confidence intervals (95% CI) [22]. Using the RDS method, results presented under population proportion are weighted proportions representing the entire FSW and MSM and TGW population, respectively, in each of the three study cities while the unweighted sample proportion represents the study participants.

### Ethics

This BBS known locally as *Kauntim mi tu* (Lit: Count me too) was approved by the PNG National Department of Health’s Medical Research Advisory Committee, the National AIDS Council Secretariat Research Advisory Committee, the PNG Institute of Medical Research’s Institutional Review Board, and the Human Research Ethics Committee at the University of New South Wales (UNSW) Sydney. The protocol was reviewed according to the Centers for Disease Control and Prevention’s (CDC) human research protection procedures and was determined to be research, but CDC was not engaged in implementing the BBS.

## Results

A total of 2091 FSW participated: 673 in Port Moresby and 709 each in Lae and Mt. Hagen. Among MSM and TGW, 863 participated across the three cities: 400 in Port Moresby, 352 in Lae, and 111 in Mt. Hagen. Details of the population socio-demographics and characteristics can be found in the article by Kelly-Hanku et al. [20] and Hakim et al. [19] for FSW and MSM and TGW population, respectively. All data reported below are weighted estimates. Data for Mt. Hagen MSM and TGW was omitted in this discussion because the sample size was not sufficient to perform weighted estimate.

### TB screening

More than half of all FSW, 72.6% in Port Moresby, 52.0% in Lae, and 52.9% in Mt. Hagen, experienced at least one of the four symptoms of TB identified by WHO (Additional file 1, Table S1). Among MSM and TGW who experienced at least one of the four TB symptoms identified by WHO was 69.0% in Port Moresby and 52.6% in Lae (Table 1).

**Table 1:** MSM and TGW—TB screening and testing and TB and HIV co-infection

	Port Moresby		Lae	
	Unweighted sample proportion, %, *N* = 400	Weighted population proportion, % (95% CI), *N* = 7500	Unweighted sample proportion, %, *N* = 352	Weighted population proportion, % (95% CI), *N* = 4700
**TB screening**				
Unexplained weight loss	50.5	52.7 (46.6–58.8)	39.2	39.0 (32.3–44.3)
Cough	37.5	40.1 (34.3–45.9)	25.6	24.6 (19.1–30.0)
Fever	28.8	26.8 (21.5–32.0)	27.8	25.2 (19.9–30.0)
Night sweats	25.0	24.6 (19.4–29.7)	29.5	29.1 (23.7–34.4)
Experienced at least one of four TB symptoms	67.8	69.0 (63.1–74.8)	55.1	52.6 (46.0–58.7)
**TB and HIV testing**				
TB positive	1.0	1.0 (0.0–2.2)	1.4	1.2 (0.0–2.4)
Rifampicin-resistant TB	25.0	4.7% (0.0–7.3)	0.0	0.0 (0.0–0,0)
HIV positive	7.7	8.5 (5–11.9)	6.9	7.1 (3.6–10.3)
HIV and TB co-infection	0.0	0.0 (0.0–0.0)	0.0	0.0 (0.0–0.0)

*MSM* men who have sex with men, *TGW* transgender women, *TB* tuberculosis, *HIV* human immunodeficiency virus, *95% CI* 95% confidence interval, % percentage, *N* total sample and population

### TB testing

In Port Moresby and Lae, all FSW who were eligible for TB testing based on the WHO four-symptom TB screening algorithm consented and provided sputum for TB testing. In contrast, in Mt. Hagen, only 47% of eligible FSW for TB testing consented and were able to produce sputum. Over half (53%) of eligible FSW in Mt. Hagen who were eligible for TB testing either did not consent to test for TB or were not able to produce sputum for testing, and these participants were referred to a TB clinic for further investigations. All MSM and TGW in all three sites who were eligible for TB testing consented and were able to provide sputum for testing.

### TB prevalence

The estimated TB prevalence based on GeneXpert®MTB/RIF results among FSW was 1.2% in Port Moresby, 0.7% in Lae, and 0.2% in Mt. Hagen (Additional file 1, Table S1). No FSW was diagnosed with rifampicin-resistant TB (Additional file 1, Table S1). Among MSM and TGW, the estimated TB prevalence was 1.0% in Port Moresby with two participants (4.7%) having rifampicin-resistant TB (Table 1). TB prevalence among MSM and TGW in Lae was 1.2%, with none testing positive for rifampicin resistance TB (Table 1).

### TB prevalence rate

Among FSW, the estimated prevalence rate of pulmonary TB was 1200 per 100,000 (95% CI 100–2400) in Port Moresby, 700 per 100,000 (95% CI 100–1200) in Lae, and 200 per 100,000 (95% CI 0–500) in Mt. Hagen. Among MSM and TGW, the estimated prevalence rate is 1000 per 100,000 (95% CI 0–2200) in Port Moresby and 1200 per 100,000 (95% CI 0–2400) in Lae.

### Co-prevalence of TB and HIV

The prevalence of HIV among FSW was 15.2%, 11.9%, and 19.6% in Port Moresby, Lae, and Mt. Hagen, respectively (see Additional file 1, Table S1). Among MSM and TGW, HIV prevalence was 8.5% in Port Moresby and 7.1% in Lae, see Table 1. Based on HIV testing as part of the study, the proportion of TB-positive individuals infected with HIV were 0.1% (95% CI 0.0–0.3) in Port Moresby and 0.2% (0.0–0.6) in Lae. In Mt. Hagen, of the FSW testing positive for TB, none had HIV (Additional file 1, Table S1). Among MSM and TGW in Port Moresby and Lae diagnosed with TB, none had HIV (Table 1).

## Discussion

Until this study, no data was available in PNG on the prevalence of TB among key populations and very little on these populations in other settings. These findings are therefore important for understanding TB among key populations other than prisoners and people who inject drugs and for a high TB burden country such as PNG more generally. Our findings are particularly concerning because all cases of TB diagnosed in our study were previously undiagnosed. This suggests that there may be a gap in TB testing services in these sites generally and for key populations specifically. Targeted TB screening and testing seems warranted. Even at the lower uncertainty bounds (95% CI), our estimated prevalence rates of pulmonary TB among FSW and MSM and TGW in PNG were relatively high. For example, our estimated TB prevalence rate for FSW in Port Moresby is 1000 per 100,000 population compared to the national case notification rate of 333 per 100,000 in 2016 [15, 23]. While the data presented is concerning, this is likely to be an underestimation since extra-pulmonary TB, which is reported to account for 42% [15] of all TB cases in PNG, was not investigated as part of this study.

The high prevalence of undiagnosed pulmonary TB reported in this survey highlights the critical need to scale up TB screening and testing to be part of an enhanced HIV prevention program for key populations. Since HIV treatment services in PNG are tasked to provide TB preventative therapy to people with HIV without signs or symptoms of TB, expanding services to integrate TB screening and testing would likely result in a greater yield, especially among key populations, which this study shows are currently under served. Conversely, the findings in this study highlight the need for TB services to be sensitized to create an enabling environment for key populations at risk of TB to be screened for TB and subsequently be tested and treated. Furthermore, being supported to complete treatment in services already sensitized to issues of stigma and discriminations affecting HIV key populations may also improve treatment outcomes for TB and reduce loss to follow-ups and disease mortality.

### Limitations

*Kauntim mi tu* is the first BBS we are aware of to include TB testing. However, as the study was initially designed and powered to measure HIV and not TB prevalence, we lack the sample size to conduct analyses to determine correlates of pulmonary TB infection. This inadequacy in power also limits us to measure the burden of TB and HIV co-infection despite report showing TB to be the single largest cause of infection and death among PLHIV in PNG [1]. The rates of rifampicin resistance were also low and may also be the result of inadequate power to adequately measure resistant TB among these populations. Further, *Kauntim mi tu* was not resourced to track participant referrals for chest X-rays among those who could not produce sputum nor track TB treatment outcomes. Therefore, our results are confined to understanding pulmonary TB prevalence.

## Conclusion

*Kauntim mi tu* is the first BBS to our knowledge to include pulmonary TB testing among key populations where it provides new and much needed epidemiological data on pulmonary TB among key populations. The analysis and results show that FSW in the three cities and MSM and TGW in the two cities in this study have a high burden of undiagnosed pulmonary TB. While our prevalence rates are higher than the national notification rate of 333 per 100,000 [15] for the year 2016, it is still likely to be an underestimate of the true burden of TB among key populations because we did not measure extra-pulmonary TB. Our work reinforces the importance of including key populations in programs targeting TB active case detection, screening, treatment, and care, irrespective of HIV. Siloed approaches for HIV and TB are likely to underserve key populations. To address PNG’s TB epidemic and in the efforts to achieve elimination of TB in PNG, TB screening and testing among key populations must be prioritized and integrated into national TB programs.

## Supplementary Information

**Additional file 1: Table S1.** FSW - TB screening and testing and TB and HIV co-infection

## Data Availability

Raw data were generated in PNG by PNG Institute of Medical Research and its collaborators. Derived data supporting the findings of this study are available from the corresponding author AKH on request.

## Abbreviations

BBS: Biobehavioural survey
CDC: Centers for Disease Control and Prevention
CI: Confidence interval
FSW: Female sex workers
MDR: Multi-drug resistant
MSM: Men who have sex with men
PLHIV: People living with HIV
PNG: Papua New Guinea
RDS: Respondent-driven sampling
TB: Tuberculosis
TGW: Transgender women
STI: Sexually transmitted infection
WHO: World Health Organization

## References

[1] WHO (2019). Global tuberculosis report 2019.

[2] UNAIDS (2016). HIV prevention among key populations.

[3] WHO, CDC, UNAIDS, FHI 360 (2017). Biobehavioral survey guidelines for populations at risk for HIV.

[4] Cunha EAT, Marques M, Evangelista M, Pompilio MA, Yassuda RTS, Souza AS (2018). A diagnosis of pulmonary tuberculosis and drug resistance among inmates in Mato Grosso do Sul, Brazil. Rev Soc Bras Med Trop.

[5] Berihun YA, Nguse TM, Gebretekle GB (2018). Prevalence of tuberculosis and treatment outcomes of patients with tuberculosis among inmates in Debrebirhan Prison, North Shoa Ethiopia. Ethiop J Health Sci.

[6] Dianatinasab M, Joulaei H, Ghorbani M, Zarei N, Rezaeian S, Fararouei M (2018). Prevalence of tuberculosis in HIV-positive prisoners: a systematic review and meta-analysis. AIDS Rev.

[7] Garfein RS, Lozada R, Liu L, Laniado-Laborin R, Rodwell TC, Deiss R (2009). High prevalence of latent tuberculosis infection among injection drug users in Tijuana, Mexico. Int J Tuberc Lung Dis.

[8] Maman M, Majzoobi MM, Torabian S, Mihan R, Alizadeh K (2013). Latent and active tuberculosis: evaluation of injecting drug users. Iran Red Crescent Med J.

[9] McDaniel CJ, Chitnis AS, Barry PM, Shah N (2017). Tuberculosis trends in California correctional facilities, 1993-2013. Int J Tuberc Lung Dis.

[10] Meijerink H, Wisaksana R, Lestari M, Meilana I, Chaidir L, van der Ven AJ (2015). Active and latent tuberculosis among HIV-positive injecting drug users in Indonesia. J Int AIDS Soc.

[11] Merid Y, Woldeamanuel Y, Abebe M, Datiko DG, Hailu T, Habtamu G (2018). High utility of active tuberculosis case finding in an Ethiopian prison. Int J Tuberc Lung Dis.

[12] Xin HN, Li XW, Zhang L, Li Z, Zhang HR, Yang Y (2017). Tuberculosis infection testing in HIV-positive men who have sex with men from Xi’an China. Epidemiol Infect.

[13] Vallely A, Page A, Shannon Dias S, Siba P, Lupiwa T, Law G (2010). The prevalence of sexually transmitted infections in Papua New Guinea: a systematic review and meta-analysis. PLoS One.

[14] NACS, NDoH-PNG (2018). Papua New Guinea National STI and HIV Strategy 2018-2022.

[15] Aia P, Wangchuk L, Morishita F, Kisomb J, Yasi R, Kal M (2018). Epidemiology of tuberculosis in Papua New Guinea: analysis of case notification and treatment-outcome data, 2008-2016. West Pac Surveill Response J.

[16] Heckathorn DD (2011). Comment: Snowball versus respondent-driven sampling. Sociol Methodol.

[17] Heckathorn DD (1997). Respondent-driven sampling: a new approach to the study of hidden populations. Soc Probl.

[18] Weikum D, Kelly-Hanku A, Hou P, Kupul M, Amos-Kuma A, Badman SG (2019). Kuantim mi tu (“Count me too”): using multiple methods to estimate the number of female sex workers, men who have sex with men, and transgender women in Papua New Guinea in 2016 and 2017. JMIR Public Health Surveill.

[19] Hakim AJ, Coy K, Badman SG, Willie B, Narokobi R, Gabuzzi J (2019). One size does not fit all: HIV prevalence and correlates of risk for men who have sex with men, transgender women in multiple cities in Papua New Guinea. BMC Public Health.

[20] Kelly-Hanku A, Weikum D, Badman SG, Willie B, Boli-Neo R, Kupul M (2020). Factors associated with HIV and syphilis infection among female sex workers in three cities in Papua New Guinea: findings from Kauntim mi tu, a biobehavioral survey. Sex Health.

[21] WHO (2017). Papua New Guinea tuberculosis profile.

[22] Handcock MS, Gile KJ, Mar CM (2015). Estimating the size of populations at high risk for HIV using respondent-driven sampling data. Biometrics..

[23] WHO (2016). Global tuberculosis report 2016.

