# Supplementary material for: High prevalence of pulmonary tuberculosis among female sex workers, men who have sex with men, and transgender women in Papua New Guinea
Source: Trop Med Health. 2021 Jan 13;49:4. doi: 10.1186/s41182-020-00293-w (PMC7805114; doi:10.1186/s41182-020-00293-w)
Supplement: Supplementary file 1 — Additional file 1: Table S1. FSW - TB screening and testing and TB and HIV co-infection [file 41182_2020_293_MOESM1_ESM.docx]

**Table 1:** FSW - TB screening and testing and TB and HIV co-infection

|  | Port Moresby | | Lae | | Mt. Hagen | |
| --- | --- | --- | --- | --- | --- | --- |
|  | Unweighted  sample proportion, %  N = 673 | Weighted population proportion, %  (95% CI)  N = 16000 | Unweighted  sample proportion, %  N = 709 | Weighted population proportion, %  (95% CI)  N = 6100 | Unweighted  sample proportion, %  N = 709 | Weighted population proportion, %  (95% CI)  N = 2600 |
| TB Screening |  |  |  |  |  |  |
| Unexplained weight loss | 50.6 | 56.2 (51.0-61.1) | 40.7 | 36.6 (32.8-40.5) | 40.3 | 37.3 (33.3-40.6) |
| Cough | 33.4 | 35.5 (30.9-40.2) | 28.6 | 24.8 (21.5-28.2) | 28.6 | 25.4 (21.8-28.3 |
| Fever | 32.0 | 33.1 (28.5-37.9) | 26.9 | 24.1 (20.6-27.6) | 26.9 | 24.6 (21.0-27.6) |
| Night sweats | 27.0 | 25.9 (21.5-30.2) | 32.3 | 29.3 (25.8-32.8) | 32.3 | 29.8 (26.2-32.9) |
| Experienced at least one of four TB symptoms | 67.4 | 72.6 (68.4-76.7) | 56.8 | 52.0 (47.9-56.3) | 56.8 | 52.9 (48.4-56.3) |
| TB and HIV Testing |  |  |  |  |  |  |
| TB Positive | 1.2 | 1.2 (0.1-2.4) | 0.9 | 0.7 (0.1-1.2) | 0.1 | 0.2 (0.0-0.5) |
| Rifampicin Resistant TB | 0.0 | 0.0 (0) | 0.0(0) | 0.0 (0) | 0.0 | 0.0 (0.0-0.0) |
| HIV Positive | 14.2 | 15.2  (11.7-18.8) | 11.8 | 11.9  (9.0-14.8) | 18.8 | 19.6  (16.1-23.0) |
| TB and HIV co-infection | 0.3 | 0.1 (0.0-0.3) | 0.3 | 0.2 (0.0-0.6) | 0.0 | 0.0 (0.0-0.0) |

female sex worker (FSW), tuberculosis (TB), human immune-deficiency virus (HIV), ninety-five percent confidence interval (95% CI), percentage (%), total sample and population(N)
